# Cardiovascular autonomic function tests in an African population

**DOI:** 10.1186/1472-6823-8-19

**Published:** 2008-12-30

**Authors:** Malvin Torsvik, Amanda Häggblom, Geir Egil Eide, Erich Schmutzhard, Kaare Vetvik, Andrea Sylvia Winkler

**Affiliations:** 1 Faculty of Health, Nord Trondelag University College, Steinkjer, Norway; 2 Haydom Lutheran Hospital, Mbulu, Tanzania; 3 Department of Neurology, Medical University of Innsbruck, Innsbruck, Austria; 4 Centre for Clinical Research, Haukeland University Hospital and Department of Public Health and Primary Health Care, University of Bergen, Norway; 5 Department of Neurology, Medical University of Innsbruck, Innsbruck, Austria; 6 Institute of Medicine, Division of Haraldsplass Deaconal Hospital, University of Bergen, Norway; 7 Interdisciplinary Centre for Palliative Medicine and Department of Neurology, Ludwig-Maximilians-University, Munich, Germany

## Abstract

**Background:**

Diabetes mellitus is becoming increasingly common in sub-Saharan Africa. Autonomic dysfunction contributes to morbidity and mortality in diabetic patients. Data on autonomic dysfunction in the African population is scarce, and no reference values for standardized autonomic function tests are available. The aim of this study was to establish cut off values for five easy-to-use cardiovascular autonomic function tests that may be suitable for resource-poor settings.

**Methods:**

We recruited 276 healthy African individuals, 156 men and 120 women, aged > 20 years. Participants were tested for (1) resting heart rate (HR), (2) HR variation in response to deep breathing, (3) HR response to standing, and (4) postural changes in systolic and diastolic blood pressure (SBP and DBP). Respective cut-off values were calculated according to the 95th or 5th percentile.

**Results:**

Taking an association of the autonomic test results with gender and age into consideration, we defined the following cut-off values: resting HR (bpm) ≥ 89 for men and ≥ 97 for women; HR (bpm) in response to deep breathing ≤ 13, ≤ 11, ≤ 9, ≤ 8, and ≤ 7 for age groups 20–29, 30–39, 40–49, 50–59, and 60+ years, respectively; HR (bpm) in response to standing ≤ 14 for 20–29 years, and ≤ 11 for 30+ years; postural decreases in SBP ≥ 17 mmHg for all age groups; and postural decreases in DBP (mmHg) ≥ 2 for men and ≥ 5 for women.

**Conclusion:**

The test battery revealed cut-off values different from those measured in Caucasians. Further studies are recommended a) to assess whether these cut off values are generally applicable, and b) to establish population specific reference values for Africans.

## Background

Diabetes mellitus (DM) is increasingly common worldwide. Estimates indicate that the total number of people with DM will more than double from 171 million in 2000 to 366 million in 2030. By 2030, more than 75% of people with DM will live in developing countries. The greatest relative increase is expected to occur in countries in the Middle East, sub-Saharan Africa, and India [1]. DM in these countries is associated with higher mortality rates because of acute and chronic complications that occur early in the course of the disease [2].

Peripheral and autonomic diabetic neuropathy (DPN and DAN, respectively) are common chronic complications of DM that occur in nearly half of diabetic patients [3]. DAN in patients with diabetes is an irreversible complication, but early detection is important, because although the condition can not be reversed, intensive diabetes care may delay its further development [4].

Data on the prevalence of DAN varies according to the type of diagnostic test employed. A review of 15 studies found prevalence rates of 1%–90%, depending on which criteria were used for diagnosis [4]. The largest of these studies suggested a prevalence of 25.3% in type 1 diabetes and 34.3% in type 2 diabetes [5]. DAN was diagnosed when at least two of six autonomic function tests were abnormal. Few studies on autonomic neuropathy have been performed in Africa. In South Africa, among 50 insulin-dependent diabetic patients, a prevalence of 32% was found based on one or more abnormal autonomic function tests [6].

DAN may affect both the parasympathetic and sympathetic nervous system [4]. Ewing et al. [7] suggested utilizing a standard battery of autonomic functions tests, including heart rate (HR) variation in response to deep breathing, HR response to standing, postural changes in blood pressure (BP), the Valsalva manoeuvre, and sustained handgrip. The standard reference values of these tests were derived from Caucasian populations. However, possible genetic and environmental factors may influence HR and BP regulation [8,9]. In a study of 207 healthy young Asian, African, and Caucasian individuals, Africans had a mean arterial BP increase in response to standing, whereas Asians and Caucasians had a decrease in mean arterial BP [10]. In Nairobi, Kenya, 325 Luo migrants displayed higher mean systolic and diastolic BP compared with 267 control Luos living in rural areas, likely demonstrating a difference in BP due to differences in rural and urban lifestyles and diet [11]. Thus, genetic and environmentally caused variations in autonomic nervous regulation may exist; hence, studying responses to autonomic function tests in an indigenous African population is highly warranted. The aim of the study was to establish reference values for a battery of five easy-to-use, non-invasive cardiovascular autonomic function tests for clinical use in indigenous Africans.

## Methods

### Setting

The study was conducted in August-December 2004 in the diabetes outpatient clinic at Haydom Lutheran Hospital, a mid-size rural hospital in the Manyara region of northern Tanzania. The clinic cares for approximately 150 diabetic patients.

### Participants

A total of 328 participants were voluntarily recruited from among hospital workers, healthy relatives of patients, and people living in nearby villages. All of the participants underwent neurological examination, with special emphasis on muscle strength; sensation in response to light touch, pinprick, monofilament, and vibration; joint position awareness; and knee and ankle reflexes. Participants were asked about symptoms of burning, tingling, numbness, dizziness, and fainting. None of the participants was on any medication at the time of study. Exclusion criteria were neurological findings (*n *= 0), hypertension (BP ≥ 160/95; *n *= 45), known addiction to alcohol (*n *= 0), anaemia (*n *= 0), and chronic diseases (DM, renal failure, tuberculosis, asthma, chronic obstructive pulmonary diseases, and peptic ulcer; *n *= 5) [12,13]. In addition, two participants were excluded because of electrocardiographic (ECG) error. Thus, at the end, the prospective study comprised 276 patients (156 men and 120 women).

### Test procedure

The test battery consisted of five autonomic function tests conducted in the following order: (1) resting HR, 2) HR variation with deep breathing, (3) HR response to standing, (4) postural changes in systolic BP, and (5) in diastolic BP measured in the lying position followed by BP on standing.

#### Resting HR

Participants lay in a supine position for ten minutes. The resting HR was thereafter recorded by an ECG (Auto Cardiner FCP-2201 Fukunda Denishi, Tokyo, Japan) for one minute.

#### HR variation with deep breathing

Participants lay in a supine position for five minutes. HR variation with deep breathing was determined by ECG recording the maximum and minimum HR for six breathing cycles. The mean difference of the maximum-minimum HR was calculated.

#### HR response to standing

Participants rested in a supine position for five minutes and then were asked to stand up unaided. The HR just before standing up and 15 seconds after standing was recorded twice from an ECG and the average was calculated.

#### Postural changes in systolic and diastolic BP

We used an automatic BP machine (Tensoval, Hartmann, Germany) that was calibrated against a standard mercury sphygmomanometer to measure BP. The lying BP was recorded after at least ten minutes of resting in a supine position. The average of two readings was recorded as the resting BP. After standing up unaided, readings were repeated after one, three, and five minutes. The lying BP was compared with the lowest standing BP and the postural change in BP was calculated.

### Statistical analysis

Assumptions of normality were investigated graphically and with Kolmogorov-Smirnov's test, and when significant the distribution of logtransfomed variables was also checked in the same way. None of the test scores were distributed normally. Hence, nonparametric tests were used for statistical analysis. The Mann-Whitney U test was used to examine the difference between two groups, and the Kruskal-Wallis test was used for differences among more than two groups. Analysis of variance was applied to double-check the results of the nonparametric tests. Spearman's rank correlation (ρ) was applied to test for monotone correlations between test results and age groups [14]. The 5th and 95th percentiles were used to define cut-off values for the five autonomic function tests; i.e., an abnormal test result was defined as occurring either above or below 95% of the results. SPSS 11.0 was used for the data analysis.

### Ethical considerations

Ethical clearance was obtained from the National Institute of Medical Research (NIMR) and the study was approved by the Tanzania Commission for Science and Technology (COSTECH). The study was carried out in accordance with the principles of the Declaration of Helsinki. We obtained free and informed consent of the participants.

## Results

Two hundred and seventy-six healthy participants aged 20–76 years with a mean (SD) age of 40.1 (13.9) years were included. There were 156 men and 120 women with a mean age of 39.4 (13.3) and 41.2 (14.7) years, respectively. The participants represented four ethnic groups: South Cushitic (68.5%; 189/276), Nilotic (15.6%; 43/276), Bantoid (14.9%; 41/276), and a mixed group of other tribes (1.1%; 3/276).

The resting mean (SD) systolic blood pressure (SBP) was 122 (12.6) mmHg (median 121, range 95–158), the mean diastolic blood pressure (DBP) was 75 (7.6) mmHg (median 76, range 56–94), and the mean resting HR was 67 (12.6) beats per minute (bpm; median 65, range 40–108). Table 1 presents variations of the above parameters by gender, age groups, and ethnic affiliation. SBP and DBP were higher in men than in women (*p *< 0.001 and *p *= 0.038, respectively), and HR was higher in women than in men (*p *= 0.001). A significant difference in SBP was found across the age groups (*p *= 0.039). Both SBP and DBP differed among tribes (p < 0.001 and *p *= 0.011, respectively). Bantu participants had the highest and Nilotic participants the lowest BP values.

**Table 1:** Blood pressure and heart rate at rest by gender, age, and ethnic affiliation in 276 healthy participants.

	Category	SBP (mmHg)	DBP (mmHg)	HR (bpm)
		
		Mean (SD) Median (range)		
		
Gender	Male (*n *= 156)	125 (12) 125 (95–158)	76 (7) 77 (56–94)	65 (12) 63 (40–108)
	Female (*n *= 120)	118 (12) 117 (95–158)	74 (8) 75 (56–94)	70 (13) 68 (49–107)
	*p *(Mann-Whitney)	<0.001	0.038	0.001
Age groups	20–29 years (*n *= 71)	125 (12) 123 (102–152)	74 (7) 73 (56–87)	67 (11) 65 (50–101)
	30–39 years (*n *= 72)	123 (11) 123 (99–158)	76 (7) 77 (58–88)	66 (13) 63 (40–107)
	40–49 years (*n *= 65)	119 (11) 117 (98–151)	74 (7) 75 (57–89)	65 (12) 63 (44–104)
	50–59 years (*n *= 39)	119 (14) 119 (95–156)	76 (9) 78 (56–94)	70 (12) 69 (52–98)
	60+ years (*n *= 29)	125 (17) 124 (95–158)	76 (9) 76 (58–94)	71 (16) 69 (50–108)
	*p *(Kruskal-Wallis)	0.039	0.074	0.190
Ethnic affiliation	South Cushitic (*n *= 189)	121 (12) 120 (95–158)	74 (7) 75 (56–94)	67 (13) 65 (40–108)
	Nilotic (*n *= 43)	119 (12) 119 (99–150)	74 (8) 74 (57–87)	68 (13) 65 (50–104)
	Bantoid (*n *= 41)	131 (13) 129 (110–158)	79 (7) 79 (64–94)	67 (13) 65 (44–107)
	Other (*n *= 3)	129 (14) 127 (116–144)	72 (16) 72 (57–88)	66 (12) 60 (58–79)
	*p *(Kruskal-Wallis)	< 0.001	0.011	0.989

**Figure 1:**
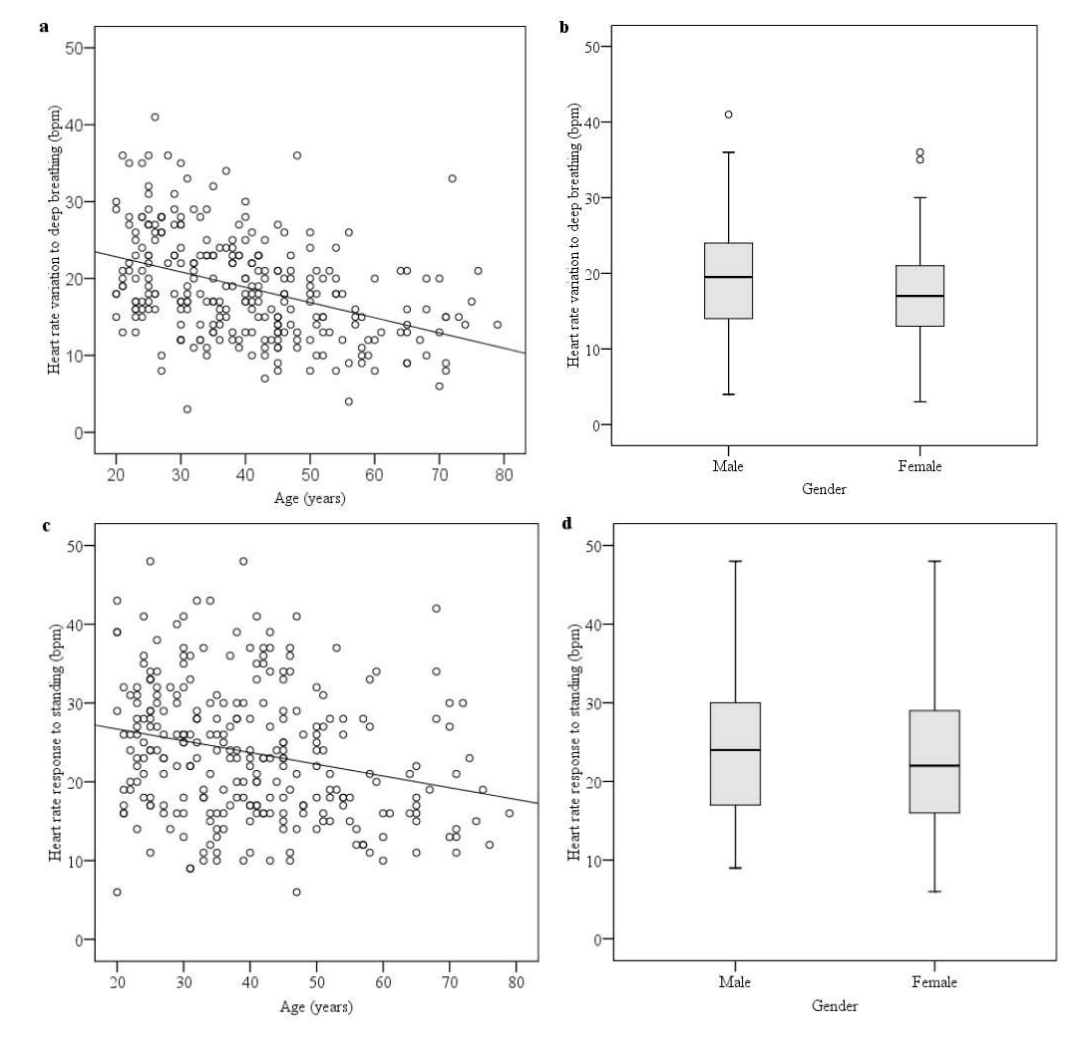
**HR variation in response to deep breathing and HR response to standing showed a significant decrease with age (a) (ρ = -0.427, *p *< 0.001) and (c) (ρ = -0.250, *p *< 0.001), respectively, but no association with gender (b and d)**.

**Figure 2:**
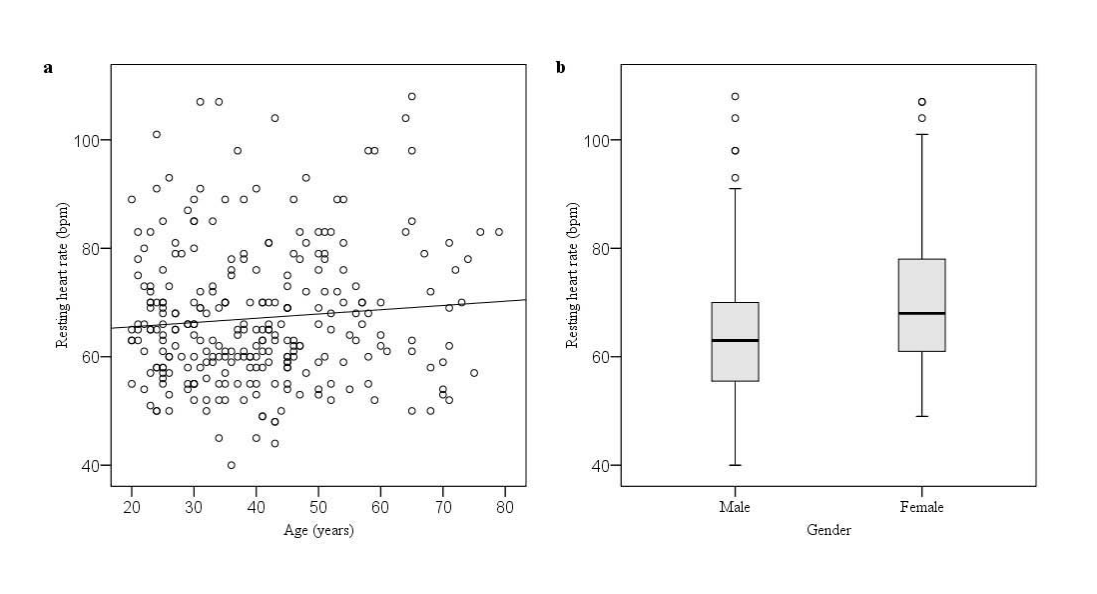
**Resting HR showed no association with (a) age (ρ = 0.047), but was significantly associated with (b) gender (*p *= 0.001, Mann-Whitney U-test)**.

**Figure 3:**
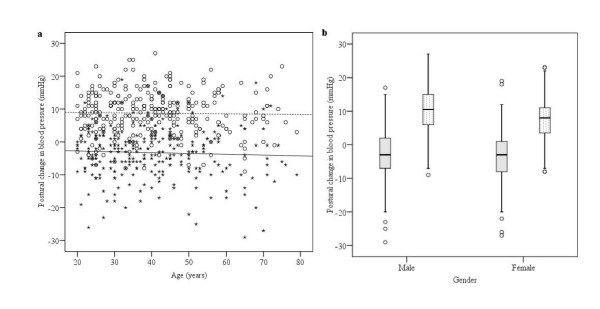
**Mean systolic BP (stars) decreased with standing in all age groups, but with no association with (a) age (ρ = -0.026, continuous line) or (b) gender (shaded bars)**. Mean diastolic BP (circles) increased with standing in all age groups, but with no significant association with age (ρ = -0.010, dotted line) (a), whereas we found a significant difference for gender (b) (*p *= 0.001, Mann-Whitney U-test, hatched bars).

When defining the cut-off values for each test in our battery, we determined the influence of age and gender on the test results. Table 2 shows whether age or gender was associated with any of the tests. We found no association with ethnic groups in any of the tests. Two of the five tests showed an association with age: HR variation with deep breathing (*p *< 0.001) and HR response to standing (*p *= 0.001). Both these tests were negatively correlated with age, (ρ = -4.23, *p *< 0.001 and ρ = -0.25, *p *< 0.001; Figure 1a and 1c, respectively). Resting HR and postural changes in DBP were associated with gender (*p *= 0.001; Figure 2b and 3b, respectively). Analysis of variance was performed as a double-check of the p-values in the non-parametric analysis, and each autonomic function test result was reciprocally adjusted for age and gender. At the 5% significance level, all of the test results remained, except for a marginally significant association of gender with HR variation in response to deep breathing, which was not significant when we controlled for age (*p *= 0.074).

**Table 2:** Age- and gender-specific mean and median values for autonomic function tests in 276 healthy participants.

Variable	Category	Resting HR (bpm)	HR variation to deep breathing (bpm)	HR response to standing (bpm)	Postural change in SBP (mmHg)	Postural change in DBP (mmHg)
		
		Mean (SD) Median (range)				
Age groups (years)	20–29 (*n *= 71)	67 (11) 65 (50.101)	23 (7) 23 (8.41)	26 (8) 26 (5.48)	-3 (7) -3 (-26.12)	8 (7) 9 (-8.23)
	30–39 (*n *= 72)	66 (13) 63 (40.107)	19 (6) 19 (335)	24 (9) 25 (9.48)	-3 (7) -3 (-20.19)	9 (7) 10 (-8.25)
	40–49 (*n *= 65)	66 (12) 63 (44.104)	18 (6) 17 (7.36)	24 (9) 23 (6.41)	-2 (7) -1 (-17.15)	10 (6) 10 (-4.27)
	50–59 (*n *= 39)	70 (13) 69 (52.98)	15 (5) 15 (4.26)	22 (7) 21 (11.37)	-4 (8) -3 (-25.14)	9 (7) 10 (-7.22)
	60+ (*n *= 29)	71 (13) 69 (50.108)	15 (6) 14 (6.33)	19 (8) 16 (10.42)	-6 (11) -7 (-29.18)	7 (7) 7 (-9.23)
*p *(Kruskal-Wallis test)		0.190	< 0.001	0.001	0.116	0.225
Gender	Men (*n *= 156)	65 (12) 63 (40.108)	20 (7) 20 (4.41)	24 (8) 24 (9.48)	-3 (8) -3 (-29.17)	10 (7) 11 (-8.23)
	Women (*n *= 120)	70 (13) 68 (49.107)	18 (6) 17 (3.36)	23 (9) 22 (6.48)	-4(8) -3 (-27.19)	7 (7) 8 (-8.23)
*p *(Mann-Whitney U-test)		0.001	0.033	0.244	0.187	0.001
All ages (*n *= 276)		67 (13) 65 (40,108)	19 (7) 18 (3.41)	24 (9) 23 (6.48)	-3 (8) -3 (-29.19)	9 (7) 9 (-9.27)

We generated cut-off values (Table 3) according to association with age or gender. The table shows cut-off values according to the 95th or 5th percentile of the five tests. Values that were greater than or equal to the cut-off values for resting HR and decreases in SBP and DBP, and less than or equal to the cut-off value for HR variation in response to deep breathing and HR response to standing, were regarded as abnormal. Gender-specific cut-off values were only computed for resting HR and postural changes in DBP, because only these tests were found to be associated with gender. Age-specific cut-off values were only computed for the tests that were found to have a significant association with age. We computed the 5th percentile cut-off values for HR response to standing as ≤ 14 (20–29 years), ≤ 10 (30–39 years), ≤ 10 (40–49 years), ≤ 12 (50–59 years), and ≤ 11 (60+ years). This test results yielded a nonlinear fall with age, which is of little clinical applicability. We found, however, a significant difference in cut-off values between the 20–29-year group and the group of participants who were 30+ years old (*p *= 0.001, Mann-Whitney U-test). Therefore, Table 3 presents cut-off values divided by these two age groups for this particular test.

**Table 3:** Cut-off values for cardiovascular autonomic tests based on ≥ 95th or ≥ 5th percentile.

Variable	Category	Resting HR (bpm)	HR in response to deep breathing (bpm)	HR in response to standing (bpm)	Fall in postural SBP (mmHg)	Fall in postural DBP (mmHg)
Age groups (years)	20–29		≤ 13	≤ 14	≥ 17^b^	
	30–39		≤ 11	≤ 11^a^		
	40–49		≤ 9			
	50–59		≤ 8			
	60+		≤ 7			
Gender	Men	≥ 89				≥ 2
	Women	≥ 97				≥ 5

The 95th percentile cut-off value applies to resting HR and postural changes in SBP and DBP.

The 5th percentile applies to HR variation in response to deep breathing and to standing.

^a^This cut-off value applies to age groups of 30+ years.

^b^This cut-off value applies to all age groups.

## Discussion

DAN usually begins with impairment of the parasympathetic nervous system, followed by damage to the sympathetic nervous system. The process is patchy, with increasing involvement of both systems [4]. A variety of tests assessing the function of both nervous systems are therefore suggested. Our study included five non-invasive and easy-to-use autonomic function tests. The Valsalva manoeuvre and sustained handgrip test were not included, even though they common autonomic function tests. The Valsalva manoeuvre may be associated with intraocular haemorrhage and dislocation of the lens [4,15], while the sustained handgrip test ideally requires a handgrip dynamometer and continuous BP recording, which often is not available in resource-poor settings. Both tests require good communication and depend on the patient's cooperation. In our test battery, we added resting HR and postural changes in DBP; two tests that are suitable for resource-poor circumstances, to the more commonly used tests of HR variation in response to deep breathing, HR response to standing, and postural changes in SBP. Reduced parasympathetic activity causes resting tachycardia, and this test is therefore a test associated with cardiovascular autonomic failure [16]. Postural changes in SBP and DBP are included in the criteria for defining orthostatic hypotension by the Consensus Committee of the American Autonomic Society and the American Academy of Neurology [17]. In autonomic function test batteries, the postural change in SBP is usually included. Postural change in DBP is used less commonly [18].

In our cohort of individuals, we found higher BP in men than in women, whereas women had a higher HR compared with men (Table 1). This is consistent with other studies. A study in adult Americans showed respective mean BPs and HRs of 122/74 mmHg and 52 bpm in men, and 113/70 mmHg and 65 bpm in women [19]. Another study from a large Caucasian French population showed higher BP in general, but with the same significant difference between men and women. The mean HR was 67 bpm in men and 70 bpm in women, which is similar to our result [20].

SBP and DBP at rest varied by ethnic affiliation with significantly higher BP among Bantoids compared with South Cushitic and Nilotic participants (Table 1). In our study population, SBP and DBP did not increase with age. In fact, SBP was lower in individuals aged 40–59 years compared with those aged 20–39 years (Table 1). We can find no comparable study with indigenous Africans to support this finding. A significant linear increase in BP with age was seen in a large study conducted in urban and rural Tanzanians in Dar es Salaam and the Kilimanjaro region, respectively [21]. Increases in BP with age are also common in Caucasians [22]. Although hypertensive participants with BP ≥ 160/95 were excluded, our results of relatively low BPs may be due to ethnic and lifestyle determinants in our study population. Epidemiological studies on BP in the African population describe a significant increase in BP when Africans move from rural to urban areas [11,23]. Our study population was rural or semi-urban, but this geographical background only partly explains our findings of relatively low BP, as a study of rural participants (*n *= 928) in the Kilimanjaro area [21] showed a mean BP in men of 129/81 and in women of 127/82, both of which were higher than our findings of BP 125/77 in men and 118/74 in women. Further studies are necessary to support our finding of a low-BP community in which BP does not seem to increase with age and to shed some more light on the possible reasons (genetic, environmental, life style) of BP differences between tribes.

The current upper range for normal HR is100 bpm [16]. This is an arbitrary value, and several studies have questioned this cut-off value as too high [24-26]. We found a significant difference in resting HR between males and females. The gender difference is consistent with other studies, and may be related to differences in baroreceptor function; thus, we defined gender-specific cut-off values for resting HR (Table 3) [19,27].

In general, established cut-off values for cardiovascular autonomic function tests in Caucasians are based on previous studies [28-30], and on the results of studies by Ewing, who reproduced and extended the test battery to five recognized non-invasive cardiovascular function tests [7]. In the literature, abnormal scores have been defined as > 2 SD below the mean and borderline results as 1.5–2 SD below the mean. This is a common way of establishing normal values in medicine besides calculating percentiles. Scores based on the SD assume normal distribution of data, which was not the case in our study. Thus, we considered results to be abnormal when the value fell outside of the 95th percentile at the upper range or the 5th percentile at the lower range.

Table 4 compares our test results with those of certain studies performed in Caucasians. HR variation in response to deep breathing was significantly lower than that of Ewing's [7] finding (*p *< 0.001), and significantly higher in the age groups 20–29 (*p *< 0.001), 40–49 (*p *= 0.001), and 70+ years (*p *= 0.001) than what was reported by Mathias and Bannister [31]. Our HR response to standing results were lower than that of Mackay et al. [30]. Although Ewing et al. [7] found a strong correlation between HR variation in response to deep breathing and age, they did not suggest age-specific cut-off values. A fixed cut-off value may cause false-positive results for younger age groups and false-negative results for older age groups. We therefore suggest using age-specific cut-off values for five different age groups when measuring HR variation in response to deep breathing, and two different age groups when measuring HR response to standing (Table 3).

**Table 4:** Comparison of autonomic function tests results of the current study with those of previous studies in Caucasians.

Age groups (years)	Autonomic function test			*p *(One-sample *t*-test)
		
	HR variation to deep breathing (bpm) (means (SD))			
		
	Current study	Mathias and Bannister [31]	Ewing et al. [7]	
		
20–29	23 (7)	20 (2)		< 0.001
30–39	19 (6)	19 (3)		0.690
40–49	18 (6)	15 (2)		0.001
50–59	15 (5)	17 (2)		0.056
60–69	14 (4)	12 (1)		0.065
70+	15 (7)	9 (1)		< 0.001
All ages	19 (7)		31 (9)	< 0.001

	HR response to standing (bpm) (means (SD))			
		
	Current study	Mackay et al. [30]		
		
All ages	24 (9)	27 (8)		< 0.001

	Postural change in SBP (mmHg) (means (SD))			
		
	Current study	Ziegler et al. [18]	Ewing et al. [7]	
		
All ages	-3 (8)	-10 (8)	-1 (8)	< 0.001^a^

	Postural change in DBP (mmHg) (means (SD))			
		
	Current study	Ziegler et al. [18]		
		
All ages	9 (7)	-2 (6)		< 0.001

^a^The *p*-value applies to both comparative studies.

We found no relationship between age and BP change on standing, which also corresponds to previous studies in Caucasians [7,31]. We found, however, a difference between men and women, with less of an increase in DBP upon standing in women (Table 2). Several studies have discussed whether women may have a less-pronounced sympathetic response to stress and therefore less vasoconstrictor activity upon standing [19,32,33], or a smaller stroke volume, leading to a lower postural BP response [34,35]. Ewing found a significant difference in the DBP response to sustained handgrips between men and women, with a more pronounced increase in DBP in men than in women, supporting the hypothesis of reduced sympathetic activity in women [7].

Our postural changes in BP results show a greater drop in mean postural SBP than was reported by Ewing et al. (*p *< 0.001), but a lower drop than was found by Ziegler et al. [7,18]. According to our calculations, we suggest that the cut-off value for abnormal drops in SBP should be ≥ 17 mmHg, and that there should be different DBP drop cut-off values for men (≥ 2 mmHg) and women (≥ 5 mmHg). These cut-off values differ from the cut-off values reported in Caucasians. Ewing defined a drop in SBP of 11–29 mmHg as borderline and ≥ 30 mmHg as abnormal. A consensus statement in 1996 defined orthostatic hypotension as a fall in SBP and DBP within three minutes of ≥ 20 mmHg and ≥ 10 mmHg, respectively [17]. Goldstein and Shapiro [10] suggested that there is an ethnic difference in sympathetic activity, peripheral artery resistance, and baroreceptor reflex response. In a study of cardiovascular changes to postural challenge in 207 healthy Asian American, African American, and Caucasian American adults, the African Americans displayed an immediate increase in mean arterial pressure (MAP) in response to standing, whereas the Asian Americans and Caucasian Americans displayed a fall in MAP. African Americans displayed greater increases in DBP in response to postural changes than either Asian Americans or Caucasian Americans. These results support our findings that African participants had less of a postural drop in SBP and a greater rise in DBP. However, the results of studies in Caucasians are not consistent (Table 4), and any conclusions should be made with caution.

In conclusion, this study is, to our knowledge, the first attempt to establish normal values for non-invasive autonomic function tests in an indigenous African population. Our cut-off values, according to the 5th and 95th percentile, suggest that there is a need for adjustments to make this group of tests an accurate diagnostic tool. However, we recommend conducting a similar study within a larger African population, preferably evenly distributed over all age ranges and of a diverse ethnic origin, to establish population specific reference values.

## Abbreviations

bpm: beats per minute; BP: blood pressure; ECG: electrocardiogram; DAN: diabetic autonomic neuropathy; DBP: diastolic blood pressure; DM: diabetes mellitus; DPN: diabetic peripheral neuropathy; HR: heart rate; MAP: mean arterial pressure; NS: not significant; SD: standard deviation; SBP: systolic blood pressure.

## Competing interests

The authors declare that they have no competing interests.

## Authors' contributions

The study was conceived and designed by AW, ES and MT. Funding was obtained by AW and ES. Data were collected by AH and AW. Data analysis and drafting of the manuscript were performed by MT. AW, ES, KV, and GEE critically revised the manuscript and made important intellectual contributions.

## Pre-publication history

The pre-publication history for this paper can be accessed here:


